# Student–Teacher Relationship: Its Measurement and Effect on Students’ Trait, Performance, and Wellbeing in Private College

**DOI:** 10.3389/fpsyg.2022.793483

**Published:** 2022-03-14

**Authors:** Li ying Bai, Zi ying Li, Wen xin Wu, Li yue Liu, Shao ping Chen, Jing Zhang, Julie N. Y. Zhu

**Affiliations:** ^1^Department of Applied Psychology, School of Humanities and Social Sciences, Fuzhou University, Fuzhou, China; ^2^College of Modern Management, Yango University, Fuzhou, China; ^3^Institute of Computing Technology, Chinese Academy of Sciences (CAS), Beijing, China; ^4^School of Economics and Management, Fuzhou University, Fuzhou, China

**Keywords:** private college, student–teacher relationship, validity and reliability testing, self-esteem, self-efficacy

## Abstract

Student–teacher relationships (STRs) have been examined by many studies. However, an omission still exists, the existing scales are not appropriate for studying STRs in private colleges because of the special character of these schools. This paper presents the development and validation of Private-College Student–Teacher Relationship Scale (PCSTRS), the first instrument to evaluate student–teacher relationships (STRs) in private colleges. The PCSTRS has six dimensions: trust, interaction, intimacy, care, approval, and comfort. In our main study, the validity and reliability of the six-factor PCSTRS model were demonstrated. The result of internal consistency coefficient indicated the high reliability of the scale, and the result of concurrent validity indicated the significant correlational relationships between the PCSTRS with other STR measures. In supplementary study, the PCSTRS was administered to 360 participants to confirm the applicability of PCSTRS and investigate the relation of STRs and students’ traits, performance, and wellbeing, as well as the differences between the private school and the public school in this relation; the analyses revealed that there were significant differences in trust, intimacy, and care between private and public colleges; positive correlations were found between STRs and self-esteem, self-efficacy, academic performance, extracurricular activity involvement, and subjective wellbeing. Present research firstly develops the PCSTRS, examined the reliability and validity, and provides the proposed nomological network among related constructs.

## Introduction

Education is the foundation of a country and has great significance to individuals and society. As an important research topic in the fields of pedagogy and psychology, STR not only reflects the life style of teachers and students, but also is a barometer of the whole education style. In the educational situations, STR plays a critical role for student outcomes, with the benefits for academic achievement, positive affect, motivation, and traits (Eccles et al., 1993, p. 342; Gehlbach et al., 2016; Lavy and Naama-Ghanayim, 2020).

STR refers to “coordinated systems of transacting components, such that both teacher and student behaviors and characteristics inform these relationships” (Pianta, 1999, 2006, 2016; Ansari et al., 2020, p. 2). Arguing that STRs are more than just interactions, (Brinkworth et al. 2017, p. 2) defined them as “teachers’ and students’ aggregated and ongoing perceptions of one another, affect toward each other, and interactions over time; these perceptions are stored in memory and guide future interactions with the other party” (p. 2).

Although many studies have examined STRs, an omission has been found in terms of the suitability of measurements for different student groups. Given the characters of private colleges and universities, the existing scale is not appropriate for studying STRs in the context of private colleges (Yee and Fruchter, 1971; Pianta and Nimetz, 1991; Faith et al., 2018; Aboagye et al., 2019). Private college education is becoming increasingly common worldwide. For example, over six million Chinese students are pursuing full-time study (Liu, 2018). It is necessary, therefore, to develop a scale to accurately measure STRs in private colleges.

## Differences Between Private and Public Education

The differences between private and public education have been widely studied in recent years (Liu, 2018; He, 2019; Connolly and Hughes-Stanton, 2020). In 2016, there were 417 private colleges in China, accounting for more than 30% of the country’s undergraduate colleges. Whereas private education had started out as a useful supplement to public higher education, it has now become an important part of higher education in China. The government has strongly supported private education, aiming to popularize higher education and improve the human resources, especially skilled manpower, required by the job market. In light of this increased focus on private education, the situation of private colleges warrants further research attention (CHSI, 2017; Liu, 2018; He, 2019; Wu and Ji, 2020).

The first main difference between public and private colleges concerns the governance structure. Private schools are supervised by a board of directors and aim to generate profit (He, 2019). Teaching staff at private schools tend to have the following characteristics: (1) Imbalanced faculty structure; in private colleges, retired teachers and young teachers account for a large proportion. The former group may tend to have more traditional educational concepts, and there may be a generational ideological gap with the students. The latter, meanwhile, are energetic but have less professional experience, potentially resulting in a shortage of experienced, competent, professional teachers (Nie, 2019). (2) Part-time teachers are an integral part of the workforce. This type of temporary employment relationship can make it difficult for these teachers to fully devote themselves to maintaining good relationships with students (Nie, 2019). (3) The stability of the teaching staff is poor. As a result of factors related to capital investment and management level, there are high turnover rates among teachers, which poses obstacles to building stable STRs (Nie, 2019).

Furthermore, with increased private college enrollment, the source of private college students tends to be complex (Li, 2019). In recent years, private college students have shown the following characteristics: distinct personality, unclear learning goals, keenness to participate in club activities, high emphasis on self-value, pursuit of material enjoyment, strong rebellious psychology, and low dependence on teachers (Nie, 2019). Additionally, due to the expensive tuition, private schools tend to attract students from more affluent socioeconomic backgrounds, who may tend to view teachers as simply providers of education (Muzika et al., 2017).

In general, the organizational structure of private schools may lead to higher job insecurity and irresponsibility among teachers. Moreover, the particularities of enrollment can lead to low dependence of students on teachers (Muzika et al., 2017; Nie, 2019). Consequently, STRs in private colleges can be characterized by little communication, utilitarianism, and emotional indifference (Liu, 2018; Nie, 2019).

Due to the differences of private and public education, it is inappropriate to measure students in private colleges with the scale specially used for measuring students in public colleges. In addition, because of the rapidly increasing number of students and some outstanding problems in private colleges, it is necessary to study STRs there, thus we are supposed to develop a tool that is more relevant to the private colleges students from their perspective.

In light of the above, the present research aims to develop and validate the Private-College Student–Teacher Relationship Scale (PCSTRS). In main study 1, we develop a preliminary framework for STRs in private colleges through semi-structured interviews. In main study 2, our proposed six-factor model of STRs in private colleges is investigated through exploratory and confirmatory factor analyses. Furthermore, concurrent validity was examined by correlating the PCSTRS with other STR measures. In supplementary study, the PCSTRS is used to investigate STRs in private colleges and study the relationship between STRs and students’ self-esteem, self-efficacy, performance, and wellbeing.

## Main Study: Development of the Private-College Student–Teacher Relationship Scale

### Main Study 1: Semi-Structured Interviews

#### Method

##### Participants

The convenience sampling method was used. We released the recruitment information of subjects on the social platform and collected 20 participants who volunteered to participate and signed the informed consent. Twenty participants from a private college in Southeastern China participated in face-to-face semi-structured interviews, including 9 males and 11 females. Data were collected from November to December 2019.

##### Procedures

In the semi-structured interviews, students were asked to recall their experiences with their teachers and describe the following as thoroughly as possible: current STRs, the main factors affecting STRs, their ideal STRs, and whether there were problems with current STRs. The semi-structured interview texts were coded by four psychology graduate students according to grounded theory and classified into different dimensions. In the coding process, four psychology graduate students ranked the importance of various aspects of the STRs according to the number of mentions in interviews.

#### Results

The results of the semi-structured interviews indicated that trust (character, emotion, and ability), initiative, communication, concern, and satisfaction were the aspects that should be included in STRs from the student’s perspective. 112 questionnaire items were compiled based on these five aspects.

### Main Study 2: Development of the PCSTRS

#### Method

##### Participants

The PCSTRS was tested using convenient sampling with the undergraduates at a private college in Southeastern China. Questionnaires were collected through an online platform which provides functions equivalent to Amazon Mechanical Turk and distributed through social platform. Participation in the study was voluntary for students and all participants provided informed consent. A total of 523 valid questionnaires were obtained (194 males and 329 females), including 90 freshmen (17.2%), 191 sophomores (36.5%), 155 juniors (29.6%), and 87 seniors (16.7%). Data were collected from November to December 2019.

##### Measures

Based on literature review and the semi-structured interview results, the proposed PCSTRS was graded at five levels. The higher the score, the better the STRs. To avoid fixed answering patterns, some items were graded in reverse.

#### Results

##### Item Analysis

Item analysis was carried out on the data. The statistical results of item discrimination showed that all items obtained significant levels (*p* < 0.01). Pearson’s correlation was used to calculate the correlation between the scores of each item and the total score. The correlation coefficients were between 0.42 and 0.76.

##### Exploratory Factor Analysis

**Figure 1:**
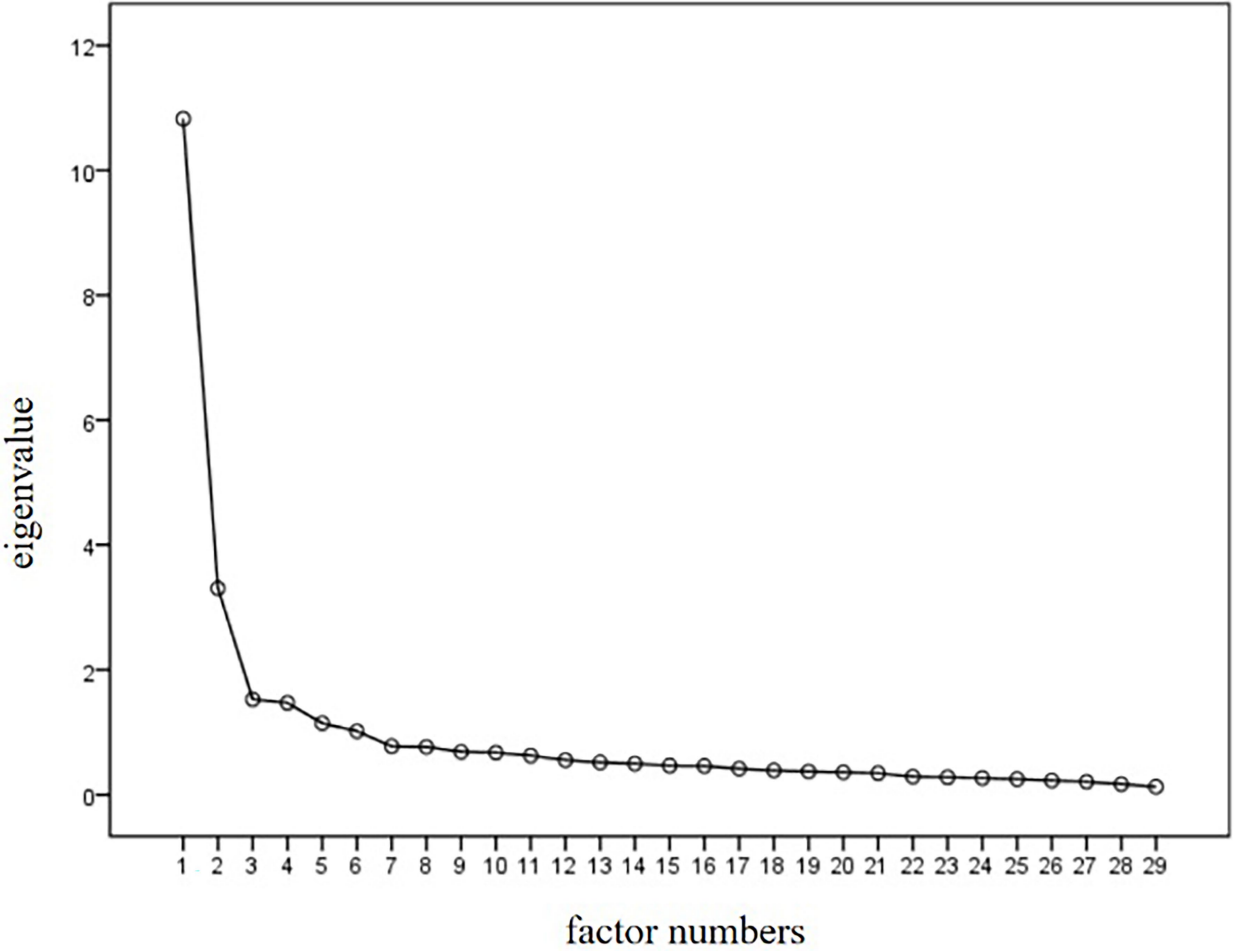
The scree plot of CFA.

The 523 observations were randomly divided into two parts: one for exploratory factor analysis (EFA; *n* = 262) and one for confirmatory factor analysis (CFA; *n* = 261). SPSS 20.0 was used for EFA. The KMO value was found to be 0.93. Bartlett’s test of sphericity showed that *χ*^2^ = 4445.24, *p* < 0.001, indicating that factor analysis was suitable (Floyd et al., 1995). Figure 1 shows the scree plot. First, principal component analysis was performed. To get the model, the characteristic root of the factor needed to be larger than 1, and the percentage of factor interpretation variance needed to be higher than 3%; further, we considered the steep order test of the scree plot and the total interpretation variance ratio. According to those indicators, we got an 18-factor model, but this model was not ideal because it was loose. A stable structure was not explored. Second, the gradual elimination method was used to explore the structure, and the items and factors were selected based on the following criteria: (1) one item cannot have a factor load on more than two factors, (2) the item’s factor load should exceed 0.4, (3) each factor cannot be less than three items, and (4) items that have very different meanings from other items of the same factor should be excluded.

After each item was removed, factor analysis was performed again using maximum orthogonal rotation. The results indicated that the six factors of our questionnaire had a clear structure and contained 29 items, which could explain 66.5% of the total variation. Table 1 shows the EFA results.

**Table 1:** The results of factor analysis (*n* = 262, 29 items).

Items	F1	F2	F3	F4	F5	F6
I have confidence in the communication skills of most teachers	0.81					
I have confidence in the expressiveness of most teachers	0.81					
I believe that most of teachers have rich social experience	0.80					
I have confidence in the teaching ability of most teachers	0.80					
I believe that most of teachers have good judgment	0.79					
My relationship with my teachers is friendly and equal	0.79					
I have confidence in the organizational ability of most teachers	0.77					
I have confidence in the guidance of most teachers	0.77					
I believe that most teachers have a wealth of teaching knowledge	0.77					
I did not interact with my teachers in class		0.84				
My teachers seldom pay attention to me in class		0.82				
I do not have much contact with teachers outside class		0.69				
I have little contact with my teachers except when necessary		0.64				
We will invite our teachers to go out and play with us			0.73			
Teachers and we have a variety of daily communication activities (such as eating and traveling), closer our relationship			0.71			
I always want to be with my teachers, not be apart			0.64			
I will keep in touch with my teachers after graduation			0.61			
My teachers cared for me and helped me to relieve the pressure in my life or in my mind			0.43			
I know the character of most of my teachers				0.80		
When I was ill, my teachers will pay attention to me				0.71		
Our teachers often give us useful instructions both in emotional and psychological aspects				0.69		
I feel very close to my teachers				0.62		
It is troublesome to make an appointment with my teachers					0.74	
Teachers come to us only when they have something needed us to do					0.67	
In order to maintain their image in the eyes of students, teachers sometimes tell lies					0.67	
Our teachers seldom have a heart-to-heart talk with us					0.65	
I never feel constrained in my relationship with my teachers						0.71
When my teachers asked me questions in class, I was happy						0.70
I like to share my experience with my teachers						0.54
Explanatory variance (total 66.5%)	37.33	11.40	5.27	5.07	3.95	3.50

Based on the factor analysis results and the implied meanings of items with high load values, the six factors were labeled as follows: F1 as trust, F2 as interaction, F3 as intimacy, F4 as care, F5 as approval, and F6 as comfort.

##### CFA

**Figure 2:**
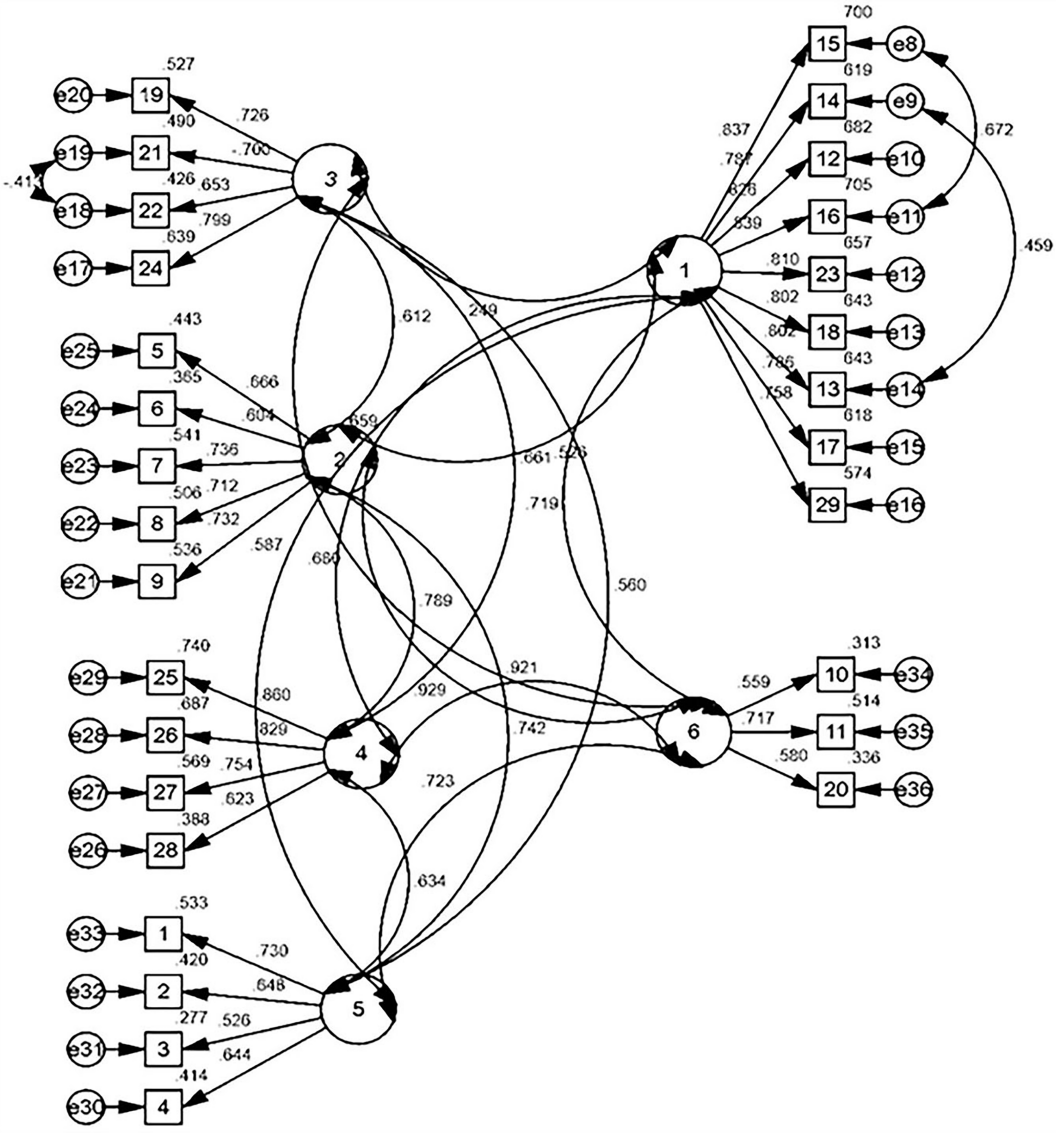
Confirmatory analysis path diagram of STRs in private colleges.

To verify the appropriateness of the six-factor model, 261 observations were analyzed using Amos 20.0 for CFA. Based on the results, six confirmatory fitting indexes of the six-factor model were obtained. The model was revised to obtain the final six-factor model for private college STRs. Figure 2 shows the final model.

The CFA fitting indexes show that *χ*^2^/*df* was lower than 5; NFI was close to 0.9; CFI, IFI, and TLI were higher than 0.9; and RMSEA was lower than 0.08. All fitting indexes were in accordance with the standard (Zhao et al., 2010; Filiz and Kaya, 2013), indicating that the model has good construction validity.

##### Reliability

The internal consistency (Cronbach’s alpha) of the questionnaire was appropriate: 0.94 for the total and 0.80–0.96 for the six factors, indicating good internal consistency (Kristine et al., 2016).

##### Validity

The correlation method was used to estimate the validity of our questionnaire. The selected criterion was the revised Student–Teacher Relationship Scale (STRS) by Pianta and Nimetz (1991) and Qu et al. (2004). With the STRS score as the calibration standard, bivariate Pearson’s product–moment correlation analysis was performed on our scale. The results showed that the developed scale was significantly correlated with the STRS (*r* = 0.82, *p* < 0.001). Moreover, the six factors of trust, interaction, intimacy, care, approval, and comfort were significantly correlated with the STRS, with correlation coefficients from 0.19 to 0.70. This shows that the criterion-related validity of our questionnaire was appropriate. According to CFA results, *χ*^2^/df was lower than 5; NFI was close to 0.9; CFI, IFI, and TLI were higher than 0.9; and RMSEA was lower than 0.08, indicating that the model has good construction validity.

Then, we retested our 29-item questionnaire using a broader range of participants from the same private school through convenience sampling, including 2953individuals (920 males, 1,539 females), and including 746 freshmen (30.3%), 614 sophomores (25%), 626 juniors (25.5%), and 473 seniors (19.2%). Cronbach’s alpha was found to be 0.94. The results showed significant differences between males and females. Specifically, males had significantly higher STR scores (95.74 ± 17.69) than females (93.56 ± 16.96; *p* < 0.05, effect size of Cohen *d* = 0.13); they also scored higher in the dimensions of intimacy, care, comfort, and interaction. There were also significant differences in STRs by grade level. The STRs of junior students (97.70 ± 19.61) were significantly higher than those of freshmen (93.40 ± 16.95), sophomores (93.07 ± 15.92), and seniors (93.23 ± 15.52; *p* < 0.05). No significant differences were found between freshmen, sophomores, and seniors.

#### Discussion

This study developed the PCSTRS based on semi-structured interview results. Through EFA, we found that the items of questionnaire can be loaded on six dimensions as we theorized (i.e., trust, identity, intimacy, care, interaction, and comfort). The result showed that the scale had good reliability and validity. Using large-sample measurement, we found there were gender and grade differences in STRs in private colleges.

## Supplementary Study: Nomological Network of STRs

This study had two major purposes. The first was to confirm the applicability of the developed scale to the private college student group. Therefore, we attempted to apply the scale developed in main study in a private school and public school to examine the differences in STRs between them. The second purpose was to investigate the relation of STRs and students’ traits, performance, and wellbeing, as well as the differences between the private school and the public school in this relation.

Given the above-mentioned differences of public and private schools, we posit:

*Hypothesis 1:* There are differences in STRs between private and public schools.

### Effect of STRs on Students’ Self-Esteem and Self-Efficacy

Self-esteem and self-efficacy are two major self-evaluative traits that have been studied widely in social psychology (Gecas and Viktor., 1989; Rosenberg et al., 1995; Burger et al., 2020) and educational psychology (Pandya, 2020). Self-esteem is considered as a core concept of individuals’ feelings about themselves, reflecting their evaluations and perceptions of themselves (Rosenberg et al., 1995). People with high self-esteem like themselves and believe they have value and importance for others (Orth and Ulrich., 2017). And, self-efficacy is a judgment of one’s capacity to achieve goals in the face of difficulty (Wang et al., 2001; Bandura et al., 2005). Students are more likely to achieve their goals when they believe they have the capacity to enact the behaviors needed to attain them.

Previous studies have revealed the positive effect of STRs on students’ self-evaluative traits (Lavy and Naama-Ghanayim, 2020). Interaction, care, sensitivity, and emotional responsiveness on the part of teachers support students’ positive self-evaluations, helping students feel more valuable, confident, and thus more likely to achieve goals (Carroll et al., 2009; Lavy and Naama-Ghanayim, 2020). We propose, therefore, that STRs will have a positive relationship with students’ self-esteem and self-efficacy:

*Hypothesis 2:* A higher level of STRs will significantly predict a higher level of (a) self-esteem and (b) self-efficacy.

### Effect of STRs on Students’ Performance

Previous studies have shown that students with better STRs tend to do better in school, such as higher academic achievement and higher participation in social activities (Carroll et al., 2009; Gehlbach et al., 2016; Ansari et al., 2020; Cui et al., 2020). Many theories have been used to explain this association. According to social motivation theory, students with high social support from teachers will build strong motivational beliefs that will promote active learning engagement and effort (Furrer and Skinner, 2003; Cui et al., 2020, p. 2). Self-determination theory links STRs, motivational beliefs, and learning behaviors, suggesting that positive relationships serve as external sources of motivational adjustment that contribute to active learning behaviors (Deci and Ryan, 2008; Ryan, 2012; Cui et al., 2020, p. 2). Furthermore, students’ perceptions of teachers in relational dimensions, such as fairness and high expectations, predicted students’ goals, academic motivation, and ultimately academic performance (Wentzel, 2010; Gehlbach et al., 2016).

In addition to academic motivation, interaction and intimacy between students and teachers can also affect students’ extracurricular performance (Hess, 2018). Affirmation, support, and organized interaction provided by teachers—also known as teacher interactional quality—have beneficial effects on students’ extracurricular participation (Roorda et al., 2011; Hess, 2018). Studies have found that an open classroom climate can nurture positive interpersonal STRs and then further strengthen students’ willingness to cooperate, take responsibility, and share (Roorda et al., 2011; Manganelli et al., 2015). Thus, we posit:

*Hypothesis 3:* A higher level of STRs will significantly predict a higher level of (a) academic performance and (b) extracurricular activity involvement.

### Effect of STRs on Students’ Wellbeing

Research in recent decades has consistently identified STRs as a key contributor to students’ wellbeing (Koster et al., 2005; Poulou, 2020). Wellbeing is defined in different ways, typically including reference to individuals’ happiness, life satisfaction, and positive affect (Campbell et al., 1976; Diener, 1984).

Researchers have suggested that care from others is a critical indicator of wellbeing (Noddings, 1984; Lavy and Naama-Ghanayim, 2020). Empirical studies have provided supporting evidence, showing that positive and stable interpersonal relationships are also important predictors of wellbeing (Seligman, 2011; Lavy and Naama-Ghanayim, 2020). Therefore, students with good, stable STRs are likely to feel more satisfied and happier, and their perception of being cared for mediates this relationship (Lavy and Naama-Ghanayim, 2020).

Apart from caring, the way in which teachers interact with students can affect students’ emotional functioning and adaptability, which subsequently influence wellbeing (Mainhard et al., 2017). The more teachers interact with students, and the better the interaction, the more students are willing to talk to teachers to deal with their negative emotions and overcome difficulties, which are conducive to happiness. Thus, we posit:

*Hypothesis 4:* A higher level of STRs will significantly predict a higher level of subjective wellbeing.

As mentioned above, there are differences in STRs between private universities and public universities. Compare to public school, the particularities of enrollment and training mode lead to the different student traits, performance, positive affect in private schools. Given the difference of STRs and student outcomes, we posit:

*Hypothesis 5:* There are differences in the relation of STRs and students’ traits, performance, and wellbeing between private and public schools.

### Method

#### Participants

The convenience sampling method was used. Questionnaires were collected through an online platform which provides functions equivalent to Amazon Mechanical Turk and distributed through social platform. Participation in the study was voluntary for students and all participants provided informed consent. The participants in supplementary study included 106 individuals (46 males, 60 females) from public colleges and 254 individuals (107 males, 147 females) from private colleges. Data were collected in November 2020.

### Measures

#### Student–Teacher Relationships

This study used the PCSTRS, using five-point Likert scales (1 = strongly disagree to 5 = strongly agree). The students were asked to think of a teacher and answer questions. It is a 29-item questionnaire encompassing six dimensions: trust, interaction, intimacy, care, approval, and comfort. Cronbach’s alpha was 0.91.

#### Self-Esteem

Self-esteem was assessed using Rosenberg et al.’s (1995) self-esteem scale (SES). It is a widely used 10-item self-report measure of self-esteem, rated from 1 = strongly disagree to 4 = strongly agree, with five reverse-scored items (items 3, 5, 8, 9, and 10). In this scale, a higher score indicates higher self-esteem. For the present sample, Cronbach’s alpha was 0.77.

#### Self-Efficacy

Self-efficacy was measured using a revised version of the general self-efficacy scale (GESE; Zhang and Schwarzer, 1995). It is a self-report questionnaire with 10 items using four-point Likert scales (from 1 = “not at all true” to 4 = “definitely true”). In this study, Cronbach’s alpha was 0.93.

#### Performance

Student performance included academic performance and extracurricular activity involvement. The former was assessed by three items: “I have done well in this course”; “In this course, I can finish my homework on time”; and “My overall performance in this course is very good” (α = 0.94). The students were asked to think of the teacher mentioned above and answer the questions based on the lessons he/she taught. The latter was assessed by inviting students to rate their participation in extracurricular activities (e.g., club activity) in the previous semester from 1 (completely inactive) to 7 (completely active).

#### Subjective Wellbeing

Students’ subjective wellbeing was measured using the subjective wellbeing index scale (WBIS) by Campbell et al. (1976) and Li and Zhao (2000). This is a self-report questionnaire using seven-point Likert scales. It is made up of two parts: index of general affect (eight items) and index of life satisfaction (one item). In this study, Cronbach’s alpha was 0.84.

### Results

In an attempt to empirically assess the potential problematic nature of common method variance in this research, Harman one-factor tests were conducted in our Study. The results suggested that common method variance does not appear to be a serious problem in this research, the variance explained by the first factor was 23.26%, less than 40%.

There were significant differences in the dimensions of trust (*t* = 2.87, *p* < 0.001, effect size of Cohen *d* = 0.33), intimacy (*t* = −2.02, *p* = 0.04, effect size of Cohen *d* = 0.24), and care (*t* = −2.54, *p* = 0.01, effect size of Cohen *d* = 0.29). Students’ trust in teachers was significantly higher in the public college (35.24 ± 6.97) than in the private college (32.95 ± 6.85). Meanwhile, intimacy and care were significantly lower in the public college (12.09 ± 4.90; 11.41 ± 3.66) than in the private one (13.25 ± 4.97; 12.47 ± 3.61), consistent with Hypothesis 1.

Table 2 shows the descriptive statistics and correlation coefficients. Table 3 shows the results of the regression analyses. After controlling for gender, age, and major, a higher level of STRs significantly predicted a higher level of self-esteem (*β* = 0.17, *p* < 0.01) and self-efficacy (*β* = 0.22, *p* < 0.001), consistent with Hypothesis 2. A higher level of STRs also significantly predicted a higher level of academic performance (*β* = 0.34, *p* < 0.001) and extracurricular activity involvement (*β* = 0.25, *p* < 0.001), consistent with Hypothesis 3. Lastly, a higher level of STRs significantly predicted a higher level of subjective wellbeing (*β* = 0.12, *p* < 0.05), consistent with Hypothesis 4. And the R^2^ of STRs on performance was much higher than that of STRs on traits and subjective wellbeing.

**Table 2:** Means, standard deviations, and correlations among variables.

	*M*	*SD*	1	2	3	4	5	6	7	8	9	10	11
1. Trust	33.62	6.96	1										
2. Interaction	11.28	3.36	−0.07	1									
3. Intimacy	12.91	4.97	0.40^***^	0.03	1								
4. Care	12.16	3.65	0.47^***^	0.01	0.71^***^	1							
5. Approval	14.08	4.04	0.09	0.43^***^	−0.33^***^	−0.24^***^	1						
6. Comfort	9.28	2.66	0.60^***^	0.07	0.78^***^	0.71^***^	−0.16^**^	1					
7 Total	93.33	15.72	0.79^***^	0.32^***^	0.71^***^	0.73^***^	0.20^***^	0.82^***^	1				
8. Self-esteem	27.99	4.00	0.26^***^	0.13^*^	−0.09	−0.09	0.30^***^	0.03	0.17^**^	1			
9. Self-efficacy	27.56	5.41	0.19^***^	−0.11^*^	0.30^***^	0.27^***^	−0.19^***^	0.32^***^	0.23^***^	0.34^***^	1		
10. Academic	16.80	3.35	0.41^***^	−0.04	0.21^***^	0.21^***^	0.01	0.29^***^	0.34^***^	0.30^***^	0.29^***^	1	
11. Activity	5.19	1.60	0.11^*^	0.07	0.30^***^	0.27^***^	−0.13^*^	0.33^***^	0.24^***^	0.01	0.21^***^	0.26^***^	1
12. Wellbeing	8.74	2.53	0.16^**^	0.09	−0.02	0.01	0.17^**^	0.03	0.13^*^	0.29^***^	0.05	0.19^***^	0.04

*n* = 360; academic = academic performance; activity = extracurricular activity involvement; wellbeing = subjective wellbeing.

* *p* < 0.05;

** *p* < 0.01; and

*** *p* < 0.001.

**Table 3:** Results of regression analysis on trait.

	Trait							
	Self-esteem		Self-esteem		Self-efficacy		Self-efficacy	
	*β*	*SE*	*β*	*SE*	*β*	*SE*	*β*	*SE*
Gender	0.13^*^	0.05	0.13^*^	0.05	−0.08	0.05	−0.06	0.05
Grade	−0.03	0.06	−0.03	0.06	−0.01	0.06	−0.01	0.06
Major	0.06	0.05	0.06	0.05	−0.05	0.06	−0.06	0.05
School type	0.19^**^	0.06	0.19^**^	0.06	−0.03	0.07	−0.03	0.06
STRs	0.17^**^	0.05	0.17^**^	0.05	0.22^***^	0.05	0.24^***^	0.05
School type * STRs			−0.01	0.05			−0.09	0.05
*R* ^2^	0.06		0.08		0.05		0.07	

*n* = 360. In school type, 1 = public college, 0 = private college.

* *p* < 0.05;

** *p* < 0.01;

*** *p* < 0.001.

**Figure 3:**
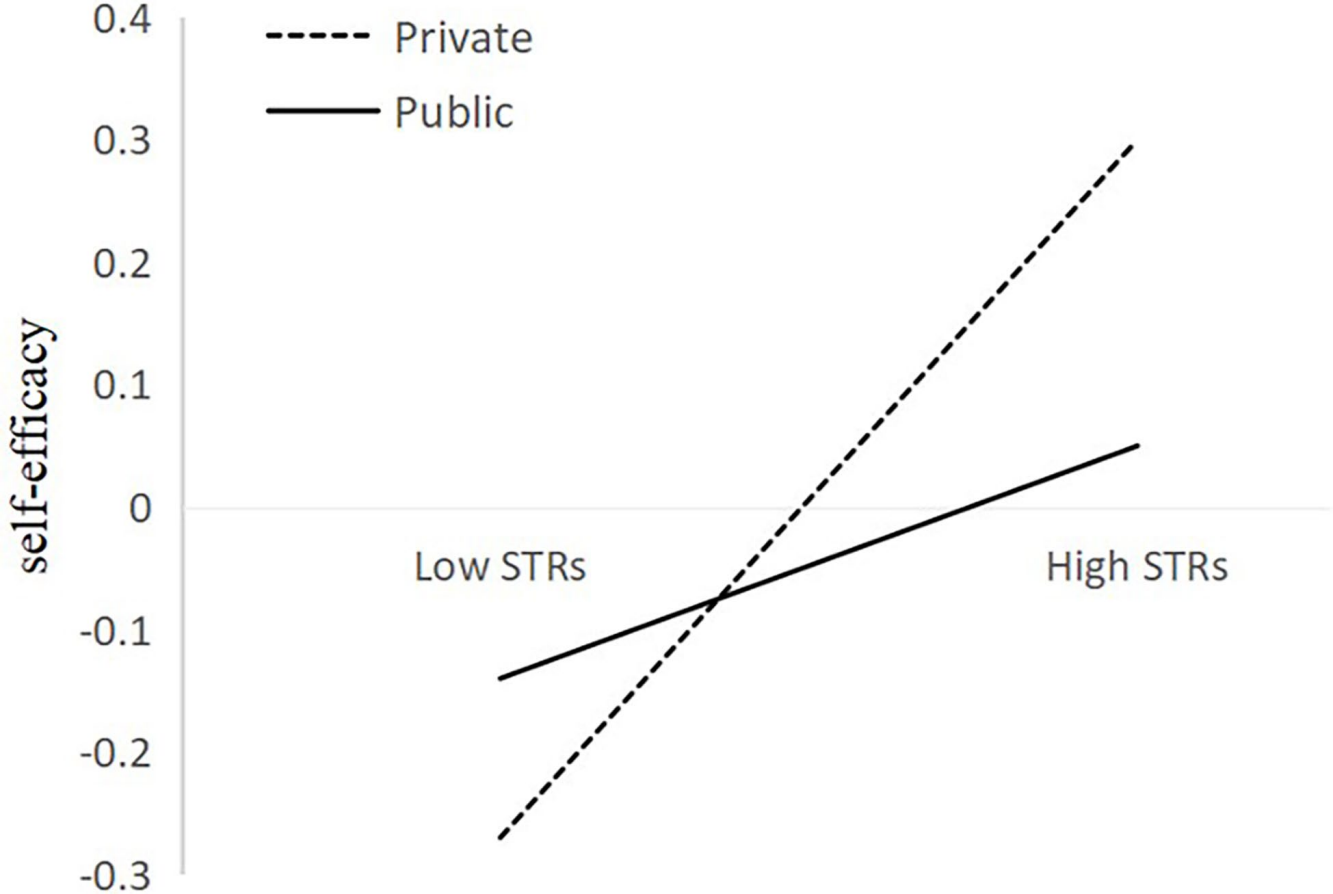
Self-efficacy predicted from STRs and moderating variables.

**Figure 4:**
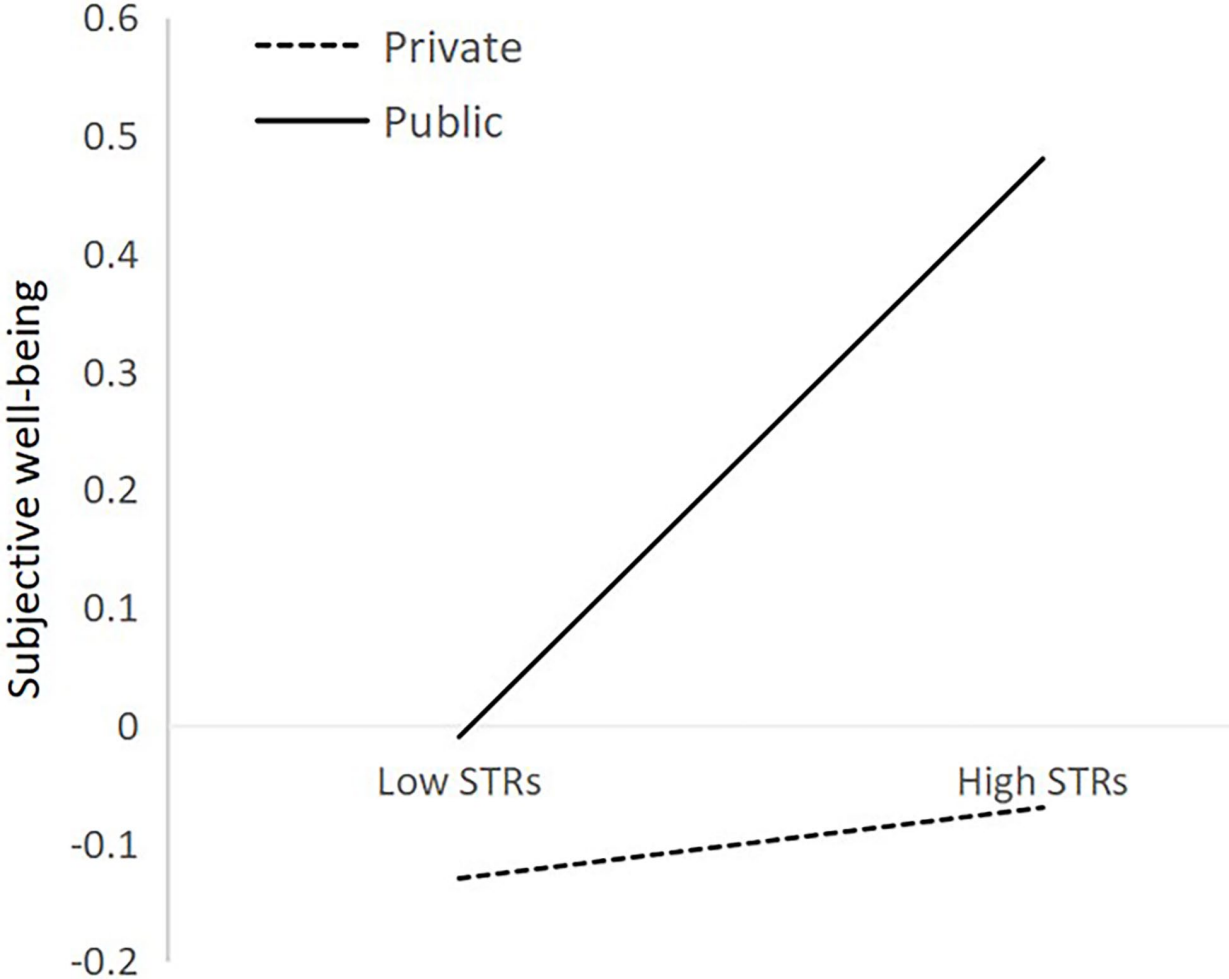
Subjective well-being predicted from STRs and moderating variables.

When examining school type factor (private or public) as moderators of the relation between STRs and self-esteem, self-efficacy, academic performance, extracurricular activity involvement, subjective wellbeing, the interaction term between STRs and school type has a significant predictive effect on subjective wellbeing (*β* = 0.10, *SE* = 0.05, *p* < 0.05) and has a marginal significant predictive effect on self-efficacy (*β* = −0.09, *SE* = 0.05, *p* = 0.07; see Tables 3, 4), supporting hypothesis 5. A higher level of STRs significantly predicted a higher level of self-efficacy in private school (*β* = 0.30, *SE* = 0.07, *p* < 0.001) than in public school (*β* = 0.10, *SE* = 0.08, *p* > 0.05) marginally. The interaction is illustrated in Figure 3. A higher level of STRs significantly predicted a higher level of subjective wellbeing in public school (*β* = 0.25, *SE* = 0.08, *p* < 0.05) than in private school (*β* = 0.03, *SE* = 0.07, *p* > 0.05). The interaction is illustrated in Figure 4. And, school type did not significantly moderate the relation between STRs and self-esteem (*β* = −0.01, *SE* = 0.05, *p* > 0.05), academic performance (*β* = 0.02, *SE* = 0.05, *p* > 0.05), extracurricular activity involvement (*β* = 0.001, *SE* = 0.05, *p* > 0.05; see Tables 3, 5).

**Table 4:** Results of regression analysis for subjective wellbeing.

	Subjective wellbeing		Subjective wellbeing		*β*	*SE*	*β*	*SE*
Gender	0.12^*^	0.05	0.10	0.05
Grade	−0.01	0.07	−0.01	0.06
Major	−0.02	0.06	−0.01	0.06
School type	0.16^*^	0.07	0.16^*^	0.07
STRs	0.12^*^	0.05	0.10	0.05
School type * STRs			0.10^*^	0.05
*R* ^2^	0.04		0.06	

* *p* < 0.05.

**Table 5:** Results of regression analysis for performance.

	Performance							
	Academic performance		Academic performance		Extracurricular activity involvement		Extracurricular activity involvement	
	*β*	*SE*	*β*	*SE*	*β*	*SE*	*β*	*SE*
Gender	−0.01	0.05	−0.02	0.05	−0.09	0.05	−0.09	0.05
Grade	0.03	0.06	0.03	0.06	−0.12^*^	0.06	−0.12^*^	0.06
Major	0.03	0.05	0.03	0.05	0.07	0.05	0.07	0.05
School type	−0.07	0.06	−0.07	0.06	−0.24^***^	0.06	−0.24^***^	0.06
STRs	0.34^***^	0.05	0.34^***^	0.05	0.25^***^	0.05	0.25^***^	0.05
School type * STRs			0.02	0.05			0.001	0.05
*R* ^2^	0.11		0.12		0.17		0.18	

* *p* < 0.05

*** *p* < 0.001.

### Discussion

In this study, by further applying the PCSTRS developed in main study, we provided evidence for the effect of STRs on students’ self-esteem, self-efficacy, performance, and subjective wellbeing. The results were similar to those of previous studies (Hess, 2018; Myrberg et al., 2019; Lavy and Naama-Ghanayim, 2020; Poulou, 2020). Comparing the STR results between a public and private college, we found differences between them in the dimensions of trust, intimacy, and care.

## General Discussion

The purpose of this paper is to develop a scale to measure STRs in private colleges, and to study the nomological network of STRs by investigating the relation of STRs and student outcomes, as well as the difference of this relation in private and public colleges. The 29-item PCSTRS developed in this research was found to have adequate psychometric properties. The six-factor dimensionality of the PCSTRS was developed and verified in main study. A high level of internal consistency was demonstrated on all subscales (αs > 0.79). With STRS scores as the calibration standard, the reliability of the PCSTRS was demonstrated in main study. Furthermore, we used this scale in supplementary study to analyze the differences between a private college and public college and identified a positive effect on students’ traits, performance, and wellbeing. The practical significance of this paper is to provide a feasible tool for studying the relationship between teachers and students in private colleges.

The particularity of STRs in private colleges was the rationale for developing a new measurement tool (Liu, 2018; He, 2019; Nie, 2019). Through EFA, we found that our PCSTRS had more dimensions than previous STR scales, including trust and interaction. We proposed that because teachers at private colleges are mainly retired teachers or young teachers, compared to public colleges, there may be differences in their specializations (Nie, 2019), teaching styles, and management abilities. Therefore, the factors of trust and approval were considered dimensions potentially worth measuring. The results confirmed that students in the public college had significantly higher trust in their teachers than those in the private college. Then, given the poor stability of teaching staff, we proposed that the interactions between students and teachers in the private college would be unique. As anticipated, the dimension of interaction also appeared in our results. In addition, this scale was developed based on the perspective of students in the private college, which can better reflect the characteristics of STRs in private schools.

In the application of our scale, we found differences by gender and grade in private college STRs, which were similar to the findings of previous studies. Men’s evaluations of STRs were significantly higher than those of women; this could be related to men’s more optimistic perceptions of relationships and positive attitudes in interpersonal communication (Fu et al., 2019). However, some have noted that STRs differences are also related to the personalities of individual students and teachers (Wang, 2019). Furthermore, the differences in grade can be largely explained by the degree of familiarity and interaction between students and teachers. Since sophomore and junior students have more curriculum tasks, they have more opportunities to have contact with their teachers and are more likely to maintain good relationships. This accords with the exposure theory of interpersonal communication.

We also found that STRs had a positive effect on students’ self-esteem, self-efficacy, performance, and wellbeing; this, too, is consistent with previous studies (Hess, 2018; Myrberg et al., 2019; Lavy and Naama-Ghanayim, 2020; Poulou, 2020). The support, interaction, guidance, and care provided by teachers can effectively promote students’ self-esteem and self-efficacy (Lavy and Naama-Ghanayim, 2020), make them feel more valuable and confident about achieving their goals (Carroll et al., 2009), improve their performance (Roorda et al., 2011; Hess, 2018; Lavy and Naama-Ghanayim, 2020; Shen et al., 2020), and increase their satisfaction and happiness (Poulou, 2020). In addition, we found that STRs were a better predictor of performance than subjective wellbeing and traits. Our results, however, suggested that the relation of STRs and students’ traits as well as wellbeing differs between public and private colleges. Specifically, although private college students reported higher scores for intimacy and care than public college students, STRs in private colleges could not promote students’ wellbeing. The reason could be that students in private colleges have more diverse sources of happiness (Songlin, 2018; Li, 2019; Nie, 2019), and their happiness does not mainly depend on the STR. They have rich entertainment and social activities, which all can boost their satisfaction and happiness (Songlin, 2018; Li, 2019; Nie, 2019), so the effect of teachers’ care and support on their wellbeing is not obvious. It is also worth noting that the STR in private colleges has a stronger tendency to predict self-efficacy than in public colleges. Self-efficacy is a judgment of one’s capacity to achieve goals in the face of difficulty (Wang et al., 2001; Bandura et al., 2005). First, generally speaking, private colleges in China have lower requirements for students’ admission scores and personal attributes than public colleges. Therefore, public college students might already have relatively stable cognitive attributes (He, 2019). Secondly, in contrast to public colleges, teachers at private schools offer students more care and support and pay more attention to students’ inner demands. In addition, the intimacy between students and teachers is significantly higher there. These characteristics of the STR may make private school students feel more empowered and more confident that they can overcome difficulties. However, student–teacher interactions at public colleges tend to be more focused on learning and academic guidance, with less attention paid to students’ personal feelings; this is an important mechanism affecting the above-mentioned individual self-efficacy. This is a complicated issue that warrants further investigation, as it may be important for revealing the effects of differences between private and public education on students, including psychological and behavioral aspects.

### Limitations and Outlook

This study has some limitations. First, the indicators used for the reliability and validity test were slightly unitary, for instance, the reliability is not discussed at the content level. It is necessary, therefore, to verify the validity of the PCSTRS using various indicators. Second, the data compiled for the scale came only from one school. Thus, the sample was not sufficiently representative, and the scale will need to be verified using a broader sample in the future. In addition, the case study could be further supplemented to clarify the characteristics of STRs in private colleges and better elucidate the functions and shortcomings of the scale.

## Conclusion

The 29-item Private-College Student-Teacher Relationship Scale (PCSTRS) developed in this research was found to have good reliability and validity. Through the investigation of STR’s nomological network, this study revealed that there were significant differences in STRs between private and public colleges, as well as the significant positive relations between STRs and self-esteem, self-efficacy, academic performance, extracurricular activity involvement, and subjective wellbeing. Furthermore, the relations between STRs and self-efficacy, wellbeing were moderated by school type (private or public). In particular, STRs were more strongly linked to students’ self-efficacy in private school than public school. In contrast, the positive correlation between STRs and subjective wellbeing was stronger among public school than private school. Present research firstly develops the PCSTRS, examined the reliability and validity, and studies the differences caused by school-running mode.

## Data Availability Statement

The raw data supporting the conclusions of this article will be made available by the authors, without undue reservation.

## Ethics Statement

The studies involving human participants were reviewed and approved by Department of Applied Psychology, School of Humanities and Social Sciences, Fuzhou University. The patients/participants provided their written informed consent to participate in this study. Informed consent was obtained from all individual participants included in the study.

## Author Contributions

LB and SC developed the study concept. Testing and data collection were performed by ZL and LL. ZL and WW drafted the manuscript, and JZ provided critical revisions. LB and ZL: These authors contributed equally to this work. On behalf of all authors, the corresponding author states that there is no conflict of interest. All authors contributed to the article and approved the submitted version.

## Funding

The work in this article was supported by the National Social Science Fund of China (No. 21BSH096).

## Conflict of Interest

The authors declare that the research was conducted in the absence of any commercial or financial relationships that could be construed as a potential conflict of interest.

## Publisher’s Note

All claims expressed in this article are solely those of the authors and do not necessarily represent those of their affiliated organizations, or those of the publisher, the editors and the reviewers. Any product that may be evaluated in this article, or claim that may be made by its manufacturer, is not guaranteed or endorsed by the publisher.
