# Supplementary material for: Student–Teacher Relationship: Its Measurement and Effect on Students’ Trait, Performance, and Wellbeing in Private College
Source: Front Psychol. 2022 Mar 14;13:793483. doi: 10.3389/fpsyg.2022.793483 (PMC8964106; doi:10.3389/fpsyg.2022.793483)
Supplement: Supplementary file 1 [file Data_Sheet_1.DOCX]

|  | Items |
| --- | --- |
| 1 | We like to chat with our teachers in our spare time |
| 2 | Teachers come to us only when they have something needed us to do |
| 3 | It is troublesome to make an appointment with my teachers |
| 4 | In order to maintain their image in the eyes of students, teachers sometimes tell lies |
| 5 | If my teachers praise me, I will think whether they are sincere |
| 6 | The relationship between me and my teacher is what I expected |
| 7 | Our teachers seldom have a heart-to-heart talk with us |
| 8 | Teachers often ask us if we need help with our study or life |
| 9 | We sometimes joke with our teachers |
| 10 | I was free to talk to the teachers and my teachers are willing to listen |
| 11 | The teachers did what they said they would |
| 12 | In general, I am satisfied with the relationship with my teachers |
| 13 | We always surround the teachers with questions and small talk |
| 14 | Teachers spend a lot of time and energy on evaluating professional titles rather than students |
| 15 | We will invite our teachers to go out and play with us |
| 16 | Teachers don't criticize me in public even if they think I'm not doing a good job |
| 17 | When I tell my teacher what I'm worried about, she doesn't think I'm worried very much |
| 18 | Associating with teachers makes me feel confident and accomplished |
| 19 | Teachers are willing to communicate with us on their initiative |
| 20 | Teacher often have little understanding of students' psychological problems except for the psychology teacher. |
| 21 | I am willing to work hard to maintain the relationship with my teachers |
| 22 | If my teacher doesn't come to class on time, I'm sure something important has happened to him/her |
| 23 | I believe that most teachers are dedicated, conscientious and responsible |
| 24 | I always want to be with my teachers, not be apart |
| 25 | Our teachers are willing to listen to us |
| 26 | Our teachers will never treat us differently because of our family background |
| 27 | The teachers cared for me and helped me to relieve the pressure in my life or in my mind |
| 28 | I believe most teachers are honest and trustworthy |
| 29 | I believe most teachers are sincere and friendly |
| 30 | Teachers and we have a variety of daily communication activities (such as eating, traveling, etc.), closer our relationship |
| 31 | My teachers are willing to share their emotional experiences |
| 32 | In getting along with teachers, many teachers do not know how to understand our thoughts and feelings |
| 33 | I will keep in touch with my teachers after graduation |
| 34 | Most teachers can be trusted |
| 35 | I believe my teachers can deal with all problems in a fair and just manner |
| 36 | Teachers often share some useful information with us through various ways (such as QQ, WeChat, face-to-face chat, etc.) |
| 37 | I am willing to open my mind to the teachers |
| 38 | If my teachers tell someone about me, it's mostly out of kindness |
| 39 | My teachers don't attract me in class |
| 40 | Teachers judge the students by scores |
| 41 | When teachers recommend books and other products to students, they seldom consider whether they are helpful to students |
| 42 | I never feel constrained in my relationship with my teachers |
| 43 | When I meet problems in study, my teachers will explain to me patiently |
| 44 | Most teachers respect and care for us |
| 45 | I find the teachers difficult to approach |
| 46 | Teachers don't pass the buck when it comes to responsibility |
| 47 | It is easy for my teachers to learn and accept new things and ideas |
| 48 | In general, I think my teacher-student relationship is harmonious |
| 49 | I like to share my experience with my teachers |
| 50 | Most teachers are tolerant and understanding |
| 51 | I often submit to my teachers' orders and authority |
| 52 | My teachers often have a keen sense of the problems in education and teaching |
| 53 | Our teachers can often handle things that difficult to us |
| 54 | I was distant from my teachers |
| 55 | My teachers and I often have different opinions and sometimes we quarrel |
| 56 | Most teachers like and care about us genuinely |
| 57 | I hope to improve the relationship between me and my teachers |
| 58 | I believe that most teachers have good interpersonal skills |
| 59 | I believe that most teachers have rich social experience |
| 60 | I feel awkward when I get along with my teachers |
| 61 | My teachers often listen to my opinions and suggestions carefully |
| 62 | My teachers were well aware of my research and were able to provide truthful and reliable information |
| 63 | I will be active in my favorite teacher's class |
| 64 | I have confidence in the organizational ability of most teachers |
| 65 | I have confidence in the expressiveness of most teachers |
| 66 | I feel a lot of pressure when I get along with my teachers |
| 67 | My teachers often make fun of me |
| 68 | My teachers care about what I say and do |
| 69 | I can accept the teachers' teaching style |
| 70 | I have confidence in the communication skills of most teachers |
| 71 | I have confidence in the teaching ability of most teachers |
| 72 | I feel there is a distance between me and my teachers |
| 73 | I can communicate well with my teachers |
| 74 | My teachers only care about the students who get good grades |
| 75 | I will not disrupt the class |
| 76 | I have confidence in the guidance of most teachers |
| 77 | I have confidence in the reflective ability of most teachers |
| 78 | My relationship with my teachers is friendly and equal |
| 79 | I don't have much contact with teachers outside class |
| 80 | Teachers seldom care about me when I am sad or wronged |
| 81 | When my teachers asked me questions in class, I was happy |
| 82 | I have confidence in the innovation ability of most teachers |
| 83 | No matter what difficulties I encounter, I believe my teachers have enough knowledge and ability to help me solve them |
| 84 | My teachers and I respect each other |
| 85 | I didn’t interact with my teachers in class |
| 86 | My teachers seldom pay attention to me in class |
| 87 | I care about my teachers very much |
| 88 | I believe that most of teachers have good judgment |
| 89 | I believe that my teachers’ decisions are correct |
| 90 | I get on well with my teachers |
| 91 | I communicate with my teachers as equals as friends |
| 92 | Teachers often don't understand my concerns |
| 93 | My teachers will squeeze time to give me guidance no matter how busy they are |
| 94 | Most teachers have a lot of theoretical knowledge, but not much practical experience |
| 95 | When I ask my teachers for professional advice, the answers are often unsatisfactory |
| 96 | I have a cold relationship with my teachers |
| 97 | I have little contact with my teachers except when necessary |
| 98 | My teachers often encourages me when I am nervous and lack confidence in answering questions |
| 99 | We will invite teachers to participate in our activities |
| 100 | I believe that most teachers have a solid foundation of knowledge |
| 101 | I believe that most teachers have solid professional knowledge |
| 102 | I feel very close to my teachers |
| 103 | I hope to get more understanding and tolerance from my teachers |
| 104 | I will take the initiative to say hello to teachers on the way |
| 105 | Our teachers often give us useful instructions both in emotional and psychological aspects |
| 106 | I know the character of most of my teachers |
| 107 | When I was ill, my teachers will pay attention to me |
| 108 | I believe that most teachers have a wealth of teaching knowledge |
| 109 | I believe that most teachers have a wealth of new knowledge |
| 110 | I hope my teachers know more about us |
| 111 | I hope to gain a lot under my teachers’ guidance |
| 112 | I believe I can graduate and get my degree successfully under the guidance of my teachers |
